# Radiograms Obtained during Anterior Cervical Decompression and Fusion Can Mislead Surgeons into Performing Surgery at the Wrong Level

**DOI:** 10.1155/2014/398457

**Published:** 2014-10-16

**Authors:** Chikato Mannoji, Masao Koda, Takeo Furuya, Yuzuru Okamoto, Tamiyo Kon, Kazuhisa Takahashi, Masashi Yamazaki, Masazumi Murakami

**Affiliations:** ^1^Department of Orthopaedic Surgery, Chiba Aoba Municipal Hospital, Aobacho 1273-2, Chuo-ku, Chiba 260-0852, Japan; ^2^Department of Orthopaedic Surgery, Chiba University Graduate School of Medicine, 1-8-1 Inohana, Chuo-ku, Chiba 260-8677, Japan; ^3^Department of Orthopaedic Surgery, Graduate School of Comprehensive Human Sciences, University of Tsukuba, 1-1-1 Tennodai, Tsukuba, Ibaraki 305-8575, Japan

## Abstract

A 68-year-old woman who suffered from C5 nerve palsy because of a C4-5 disc herniation was referred to our hospital. We conducted anterior cervical decompression and fusion (ACDF) at the C4-5 level. An intraoperative radiogram obtained after exposure of the vertebrae showed that the level at which we were going to perform surgery was exactly at the C4-5 level. After bone grafting and temporary plating, another radiogram was obtained to verify the correct placement of the plate and screws, and it appeared to show that the plate bridged the C5 and C6 vertebrae at the incorrect level. The surgeon was astonished and was about to begin decompression of the upper level. However, carefully double-checking the level with a C-arm image intensifier before additional decompression verified that the surgery was conducted correctly at C4-5. Cautiously double-checking the level of surgery with a C-arm image intensifier is recommended when intraoperative radiograms suggest surgery at the wrong level.

## 1. Introduction

Wrong-site surgery (WSS) is rare [1–15], but once it occurs, it distresses both patients and doctors [6]. Therefore, spine surgeons should make every effort to avoid wrong-site surgery. Here, we report a rare experience where a radiogram, which was obtained during anterior cervical decompression and fusion (ACDF), almost misled a surgeon into performing surgery at the wrong level.

## 2. Case Report

**Figure 1:**
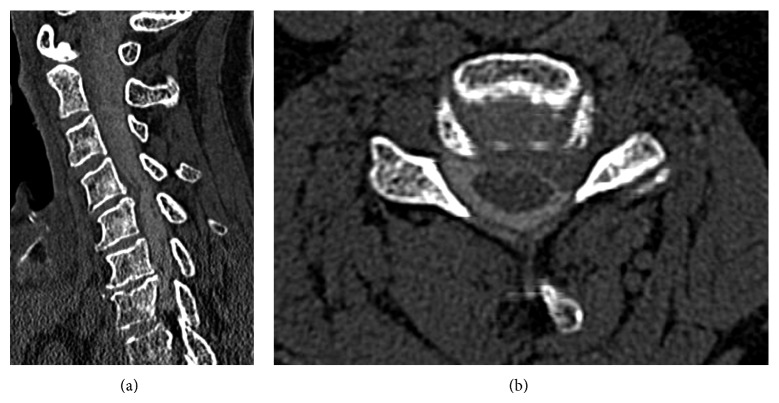
Computed tomography after myelography showing left-side C4-5 disc herniation. (a) Parasagittal view and (b) axial view at the C4-5 level.

A 68-year-old woman suffered from left-side C5 nerve palsy because of a C4-5 disc herniation. Manual muscle testing scores of her left-side deltoid and biceps were 1 and 4, respectively, and physical examination showed no symptoms of myelopathy. Magnetic resonance imaging and computed tomography (CT) after myelography showed that the herniated disc at the C4-5 level compressed her left C5 nerve (Figure 1).

**Figure 2:**
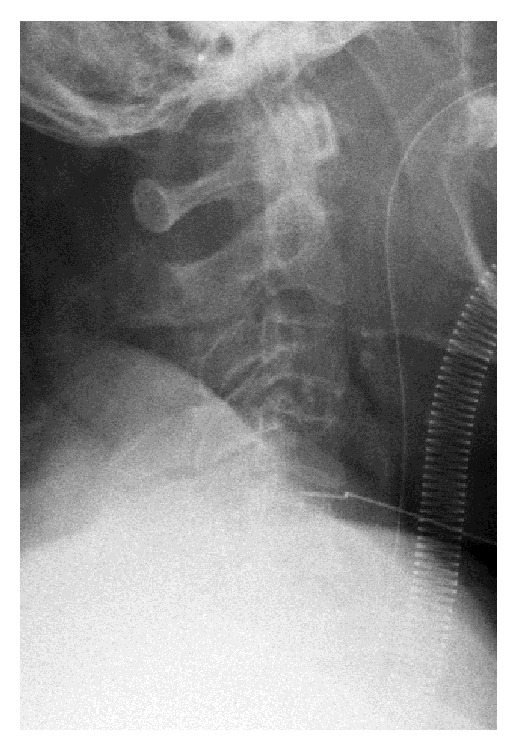
The first intraoperative radiogram after exposure of the vertebrae showing the needle inserted into the C4-5 disc.

**Figure 3:**
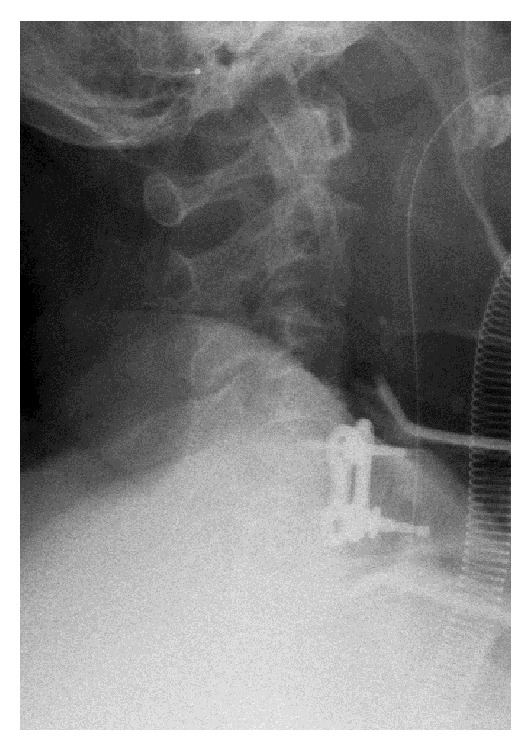
The second intraoperative radiogram after decompression, bone grafting and temporally plate fixation. It appears to show that the plate bridges the C5 and C6 vertebrae.

**Figure 4:**
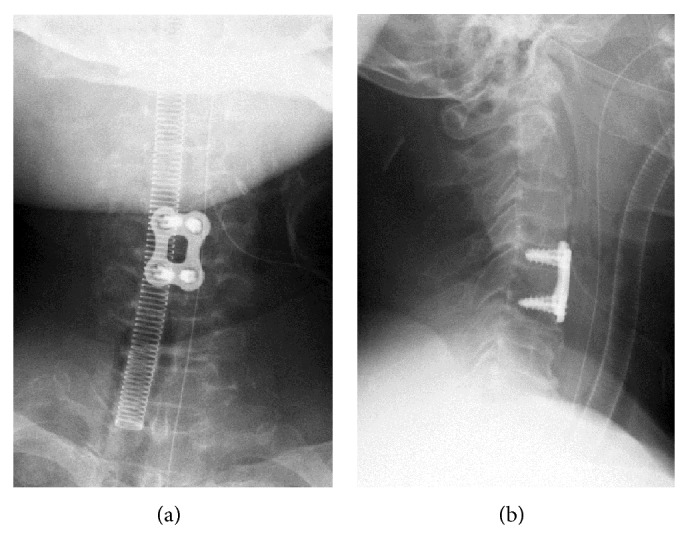
The final radiograms before extubation showed that ACDF was indeed performed at the correct level at C4-5. (a) Anteroposterior view and (b) lateral view.

We conducted ACDF at the C4-5 level. During ACDF, we always obtain two radiograms to avoid WSS. One is taken after exposure of the vertebrae, with a needle inserted into a disc to verify that the level at which the decompression and fusion are to be conducted is correct. The other one is taken after temporary fixation of a plate following bone grafting to verify the correct placement of the plate and screws. During the surgery for the current case, the first radiogram showed that the needle was inserted into the C4-5 disc (Figure 2), so we continued the surgery and performed the herniotomy and bone grafting. After bone grafting, we positioned a plate to bridge the C4 and C5 vertebrae and fixed them temporarily. The radiogram after temporary placement of the plate astonished the surgeon because it appeared to show that the plate bridged the C5 and C6 vertebrae (Figure 3). The surgeon removed the plate and was about to begin decompression of the upper level. However, because we were unable to determine the reason why the level was apparently incorrect, we decided to double-check the level with a C-arm image intensifier before decompression of the upper level. The image verified that the surgery was conducted correctly at the level of C4-5, and not C5-6 as we were mistakenly led to believe. The final radiograms before the extubation also verified that the surgery was correctly performed at the C4-5 level (Figure 4).

**Figure 5:**
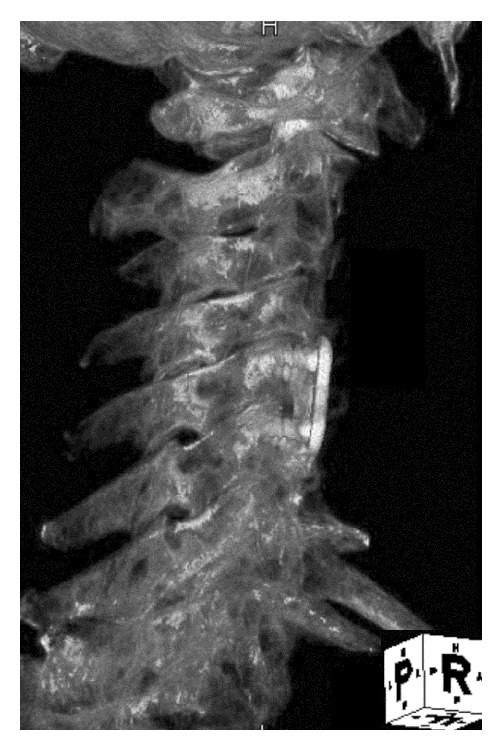
A constructed picture simulating the second intraoperative radiogram was obtained from 3D CT after the surgery. The plate appeared to bridge the C5 and C6 vertebrae.

After completing the surgery, we investigated why the radiogram apparently indicated the wrong site. Using a 3D CT image obtained after the surgery, we were able to construct a picture in which it appeared as if the plate bridged the C5 and C6 vertebrae (Figure 5). This revealed that the radiogram was taken from a caudal to cranial perspective during the surgery, and that the direction of exposure was not perpendicular to the axis of the spine.

## 3. Discussion

Various risk factors of WSS of the spine have been reported including emergency surgery, obesity, anatomic variations, time pressure to complete surgery, unusual equipment, multiple surgeons involved in the surgery, multiple procedures in a single surgery, and insufficient communication between the surgical team and the patient [10, 12, 14, 16–20]. In addition, failure to identify the vertebral level by intraoperative radiograms and misinterpretation of the radiogram are especially associated with wrong-level surgery [18, 20, 21]. As for cervical spine surgery, inadequate radiograms of the lower cervical spine hidden by the shoulders and cervical anomalies including Klippel-Feil syndrome and a block vertebra at C2-3 are major causes of wrong-level surgery [20]. In the current case, the patient did not have any of these factors.

There are some protocols for preventing WSS [22–24]. However, the effectiveness of the implementation of these protocols is controversial. Vachhani and Klopgenstein reported that the universal protocol (UP) by the Joint Commission on Accreditation of Healthcare Organizations was effective to reduce WSS events [25], but Wong and Watters III reported the UP was not effective [26]. Kwaan et al. reviewed cases and concluded that even the implementation of the UP would not have prevented 38% of WSS [8]. One of the main methods to avoid WSS is the use of radiograms during the surgery and this method is supported by many surgeons [6, 7, 14, 15, 27–29]. However, radiograms during the surgery cannot avoid every case of WSS because some patients have congenital anomaly of the spine or where radiograms are inadequate [10, 12, 25]. Some authors recommend using fluoroscopy during the surgery to identify correct levels for spinal surgery [13, 15, 30, 31]. Mayer et al. reported that surgeons now use fluoroscopy more frequently than plain radiograms during posterior surgery of the thoracic and lumbar spine, and surgeons who experienced WSS tend to have used plain radiograms more than fluoroscopy [31]. Intraoperative CT scan [32–36] is also useful to prevent WSS, but using this method routinely for only localizing the correct level is not practical.

In the current case, we obtained two radiograms during ACDF and the second radiogram almost misled a surgeon into performing unnecessary decompression at the wrong level, even though the patient did not have any anatomical anomalies of the cervical spine and the shoulders of the patient were pulled caudally during the radiograms to make it easier to see the correct level. On the other hand, a C-arm image intensifier clearly showed that we performed ACDF at the correct level. The cause of this event was that the second radiogram was inadequate and the surgeon could therefore not correctly interpret the picture. We constructed another picture from the 3D CT after the surgery that was similar to the second radiogram. This constructed picture revealed that the second radiogram was taken from a caudal to cranial perspective and the direction of exposure was not perpendicular to the axis of the spine as believed. Careful examination of Figure 5 shows that the “C3-4” disc is not clearly visualized. However, the surgeon in the operating room is under pressure to interpret radiograms quickly in less than ideal conditions and so their evaluation is compromised if they are inadequate. We highly recommend using a C-arm image intensifier to double-check the level of surgery if an intraoperative radiogram shows an unexpected finding, because a C-arm image intensifier can provide many images on many planes at once whereas plain radiograms do not offer real time feedback when the image is oblique or obscured by the shoulders. The fact that surgeons now use fluoroscopy more frequently than plain radiograms and surgeons who experienced WSS tend to have used plain radiograms more than fluoroscopy [31] also indicates that fluoroscopy is more useful than plain radiograms.

In conclusion, radiograms obtained during ACDF surgery can mislead surgeons into performing surgery at the wrong site. Cautiously double-checking the surgical level with a C-arm image intensifier is recommended when intraoperative radiograms suggest wrong-site surgery.
